# Upper tract urothelial cancer (UTUC) genomic profiling and correlation regarding benefit of platinum-based chemotherapy

**DOI:** 10.37349/etat.2024.00274

**Published:** 2024-10-17

**Authors:** Min Woo Hwang, Jasmine Kauffeld, Sarah Belay, Joep J. de Jong, Elai Davicioni, Wenping Li, Jeanny B. Aragon-Ching

**Affiliations:** IRCCS Istituto Romagnolo per lo Studio dei Tumori (IRST) “Dino Amadori”, Italy; ^1^Department of Internal Medicine, Inova Fairfax Hospital, Fairfax, VA 22031, USA; ^2^Masters of Public Health Program, University of Virginia, Charlottesville, VA 22903, USA; ^3^School of Medicine, University of Virginia, Charlottesville, VA 22903, USA; ^4^Department of Urology, Erasmus University Medical Center, 3015GD Rotterdam, The Netherlands; ^5^Veracyte Inc, Decipher Biosciences, San Diego, CA 92121, USA; ^6^Department of Pathology, Inova Fairfax Hospital, Fairfax, VA 22031, USA; ^7^GU Medical Oncology, Inova Schar Cancer Institute, Fairfax, VA 22031, USA

**Keywords:** Upper tract urothelial cancers, neoadjuvant chemotherapy, molecular subtypes, Decipher Bladder^®^

## Abstract

Upper tract urothelial cancer (UTUC) are rare subsets of urothelial cancer, which typically present with more aggressive course. Molecular markers stratifying urothelial tumors as luminal subtype and non-luminal subtype tumors have been proposed to select patients who may have greater or lesser benefit from neoadjuvant systemic therapy in bladder cancer, though not yet evaluated in UTUC. Here, a single-institution study retrospectively obtained clinical and genomic information in patients with UTUC and evaluated four patient tumors using the Decipher Bladder^®^ assay and Foundation Medicine^®^ test. All four patients had non-luminal molecular subtype including basal (*N* = 4) and mixed basal/claudin-low (*N* = 2) subtypes. The best clinical response achieved was stable disease in a patient who had basal/claudin-low subtype with residual ypT3 after neoadjuvant chemotherapy. For the remaining three patients, all were treated with platinum-based chemotherapy for eventual metastatic disease but all three showed progressive disease with limited overall survival, highlighting their aggressive course. The non-luminal subtype and lack of *FGFR* alteration may partly explain the poor overall outcomes while the real-world benefit of next generation sequencing for clinical use in UTUC patients require further clarification in a larger cohort study.

## Introduction

Upper tract urothelial cancer (UTUC) is a relatively rare manifestation of urothelial cancer (UC) that tends to be more aggressive [1]. The standard treatment for high-grade disease involves surgery in the form of radical nephroureterectomy (NU). There is also level 1 evidence on the use of adjuvant chemotherapy based on the POUT trial, which has showed improvement in disease-free survival (DFS) [2]. Additionally, a meta-analysis revealed better evidence for adjuvant chemotherapy than there is for neoadjuvant chemotherapy (NAC) at this time [3]. Although the use of cisplatin-based NAC is considered nephrotoxic and there is currently no published level 1 evidence supporting the use of NAC in the management of high-grade UTUC (HG UTUC), a neoadjuvant approach may still be beneficial prior to definitive surgery [4]. Therefore, NAC followed by surgery is often used in clinical practice [5]. Nevertheless, much of our understanding of NAC benefit in UTUC is extrapolated from bladder-UC data and from retrospective or small prospective UTUC cohort studies.

Of interest, several molecular biomarkers, such as transcriptome-based molecular subtypes, have been evaluated for use in urothelial bladder cancer. At the highest level, bladder tumors can be classified among luminal and non-luminal subtypes and non-luminal tumors have been suggested to benefit most from NAC [6]. In the present study, we assessed molecular subtyping classifications of UTUC patients who underwent NU with or without NAC, by using Decipher Bladder^®^, a clinical-grade, transcriptome-wide assay that has been validated in the peri-operative setting for bladder cancer [7]. The validated signatures include classes of Luminal, Infiltrated, Basal, Claudin-Low [8, 9] and Neuroendocrine-like (NE-like) [10, 11].

We conducted a small, single institutional, retrospective pilot study to obtain information regarding genomic profiling and molecular subtypes of four consecutive patients with a final diagnosis of UTUC who underwent NU and platinum-based chemotherapy [12]. A supplement to an existing IRB retrospective protocol was submitted to evaluate molecular subtypes in these patients. Four FFPE archival tissue samples were assayed through the commercially available Decipher Bladder^®^ test (Veracyte Inc, San Diego, CA). The tissue block with the highest grade and volume of disease was selected, and a region of interest for the assay was selected after pathological review of the hematoxylin & eosin tissue slide. The Foundation One^®^ next generation sequencing test was available for two of the four patients (Foundation Medicine, Cambridge MA). Further evaluation included clinical phenotypes of response, demographics, clinical staging, imaging results, final pathologic grade, and tumor stage. Overall survival and progression-free survival were evaluated though no formal statistical analyses were conducted given the small sample size of the study.

## Case report

**Figure 1:**
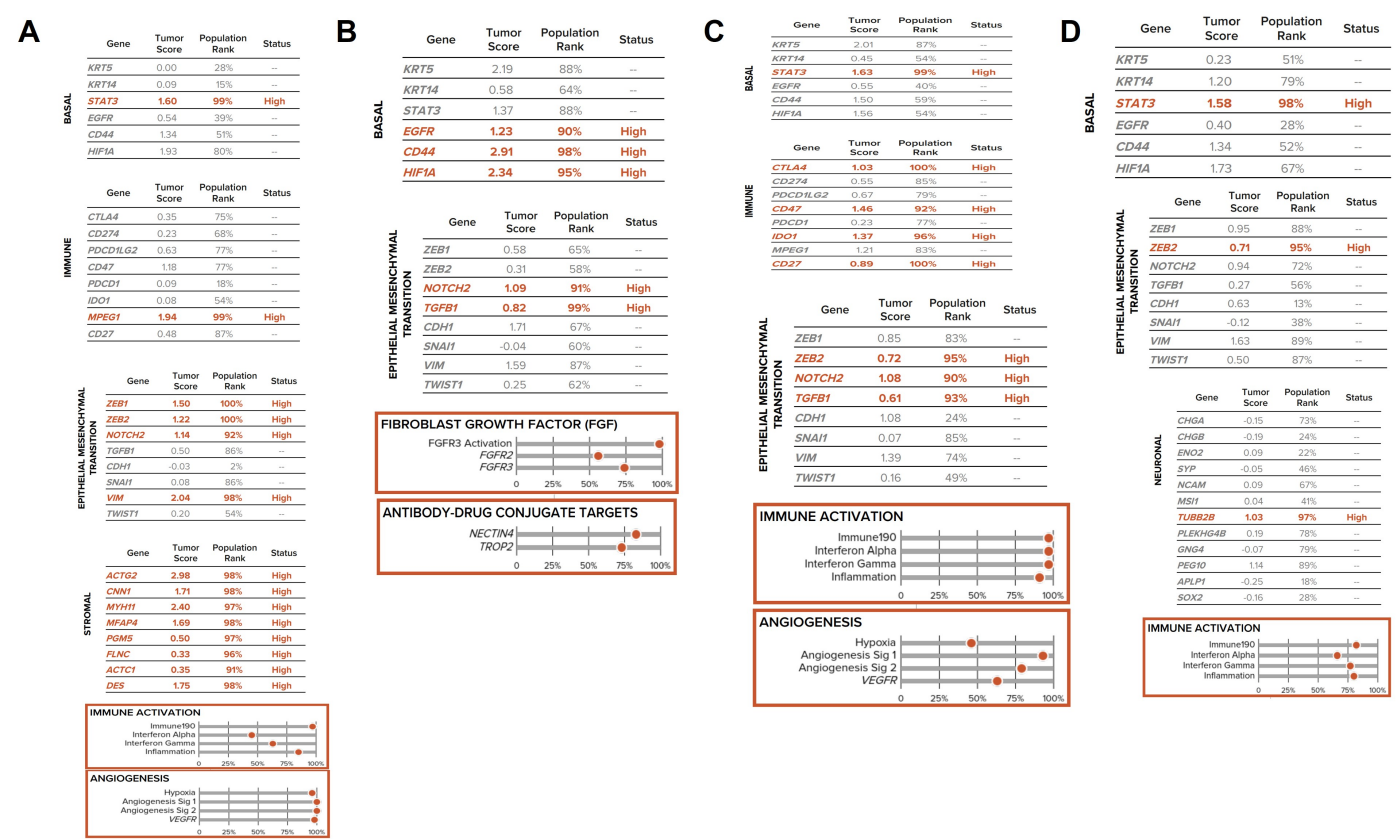
Bladder Decipher^®^ GRID. A. Pertains to clinical case 1; B. pertains to clinical case 2; C. pertains to clinical case 3; D. pertains to clinical case 4. Selected marker expression from the Bladder Decipher^®^ Grid which contains RNA expression values with the list of 48 genes grouped according to biological modules of luminal, basal, immune, epithelial-mesenchymal transition, stromal, and neuronal. The population rank refers to the percentage of tumor RNA profiles in the GRID Reference population. The bottom panel shows the genomic atlas or gene expression signatures that defines tumor biology

Four patient samples that were sent for transcriptome-wide analysis are reported herein. Table 1 summarizes clinical characteristics, and Figure 1 depicts selected marker expression from the Bladder Decipher^®^ Grid from these four cases.

**Table 1:** Clinical, genomic characteristics, and Decipher^®^ results

**Case characteristics**	**Case 1**	**Case 2**	**Case 3**	**Case 4**
Age/Sex	72/Male	57/Male	80/Female	64/Male
Upper tract location	Renal pelvis and ureter	Renal pelvis and ureter	Renal pelvis	Renal pelvis
Initial stage	III	IV	III	IV
Metastatic (Y/N); sites	N; N/A	Y; lung, bronchus	Y; RP nodes, liver	Y; spine osseous
Neoadjuvant chemo (Y/N); cycles	Y; 3	N; N/A	N; N/A	N; N/A
Treatment response	Residual ypT3 after NAC but without metastatic disease	Progressive disease	Progressive disease	Initial stable disease then progressive disease
Final clinical stage	Stage III	Stage IV	Stage IV	Stage IV
Final pT stage	ypT3	pT3cN1cM1	pT3cN2Mx	pT3aNxM1
Variant histology	HG UTUC with glandular and focal squamous differentiation	HG UTUC with squamous differentiation of 40%	Pure HG UTUC	HG UTUC with 30% sarcomatoid features and 20% necrosis
Decipher**^®^** subtype probability	Basal probability 53.4%/Basal Claudin-low 43%	Basal probability 80.4%	Basal probability 82.4%	Basal probability 39.4%/Basal Claudin-low probability 43.5%
Decipher subtype	Basal/Basal Claudin-low	Basal	Basal	Basal/Basal Claudin-low
Consensus subtype	Stroma-rich	Basal/Squamous	Basal/Squamous	Stroma-rich
UNC subtype	Basal	Basal	Basal	Basal
*FGFR3* activity class	inactive	inactive	inactive	inactive
Immune190 score	0.70	0.27	0.68	0.48
ESTIMATE stromal score	3,675	–875	1,889	2,245
Foundation medicine (NGS results)	N/A	PD-L1 TPS 1%; MS-Stable; TMB—6 Muts/Mb; CASP8 loss exons 3-7; CDKN2A loss; CDKN2B loss; HRAS G12S; MTAP loss; TERT promoter -124C>T; VUS: BTK Y418H; CDH1 V794I; CSF1R V48M; HRAS L163fs*52; MITF E207G; NOTCH3 S872F; RBM10 G280V; RPTOR D821N; SNCAIP R269Q; TSC2 R1268H	MS-Stable; TMB—11 Muts/Mb; AKT2 amplification; CDKN2A loss; CDKN2B loss; ERBB3 E332K; KDM6A Q670*; MTAP loss; RAF1 amplification; TERT promoter -124C>T; TP53 loss	N/A
Overall survival	36.6 mos	8.7 mos	10.8 mos	14.2 mos
Progression free survival	N/A	6 mos	0.7 mos	2.5 mos
Decipher gene outlier category	Immune activation/angiogenesis	FGF/ADC	Immune activation/angiogenesis	Immune activation

HG UTUC: high-grade upper tract urothelial carcinoma; mos: months; N/A: not applicable; NAC: neoadjuvant chemotherapy; RP: retroperitoneal; TPS: tumor proportion score; VUS: variants of unknown significance; UNC: University of North Carolina; NGS: next generation sequencing; PD-L1: programmed death ligand 1; TMB: tumor mutational burden; FGFR: fibroblast growth factor receptor

### Clinical case 1

**Figure 2:**
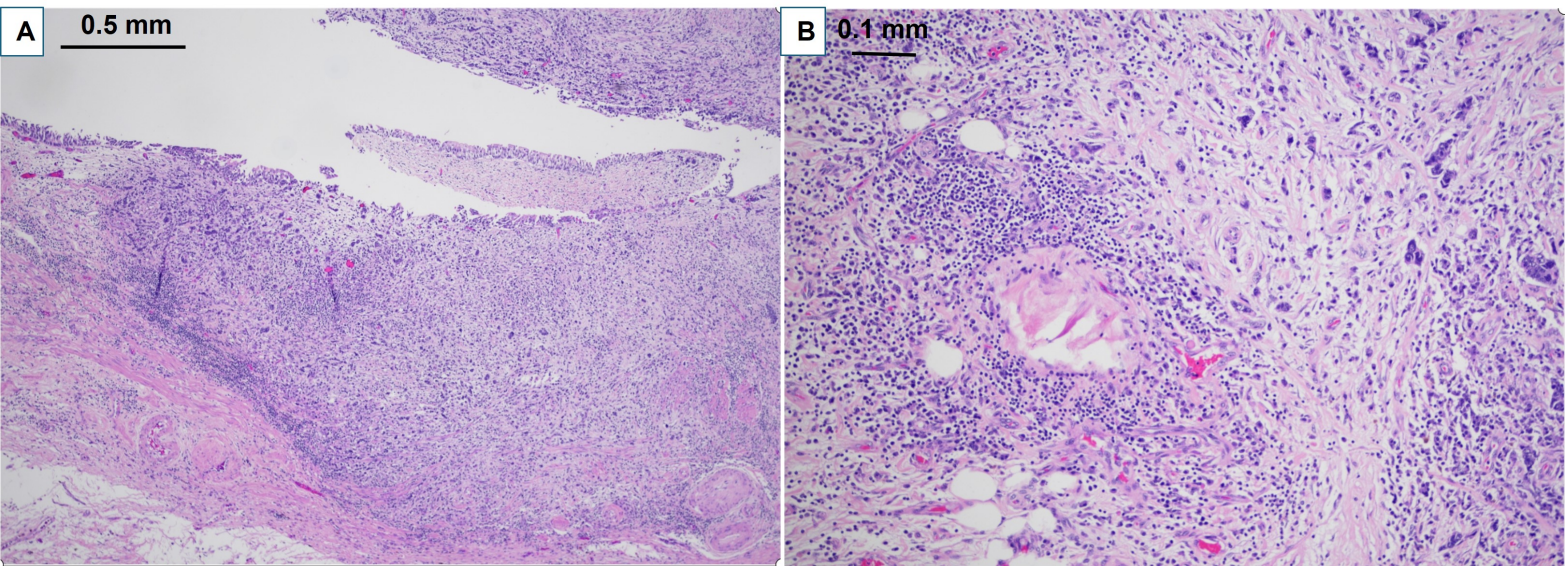
Pathological findings on H&E for clinical case 1. A. Clinical case 1 pathological staging ypT3 high grade urothelial carcinoma with glandular and focal squamous differentiation involving the renal pelvis (40× H&E); B. high grade urothelial carcinoma extending into peri pelvic adipose tissue (100× H&E)

Case 1 was a 74-year-old male with coronary artery disease presented with a left upper tract mass with positive urine cytology showing high-grade UC (HGUC). He underwent four cycles of gemcitabine and cisplatin, with imaging findings showing stable left renal pelvis mass. A radical NU was performed, and the final pathology showed ypT3pNxMx HG UTUC with glandular and focal squamous differentiation (Figure 2). While stable findings were seen without evidence of distant disease, eventual death ensued from cardiovascular disease.

### Clinical case 2

**Figure 3:**
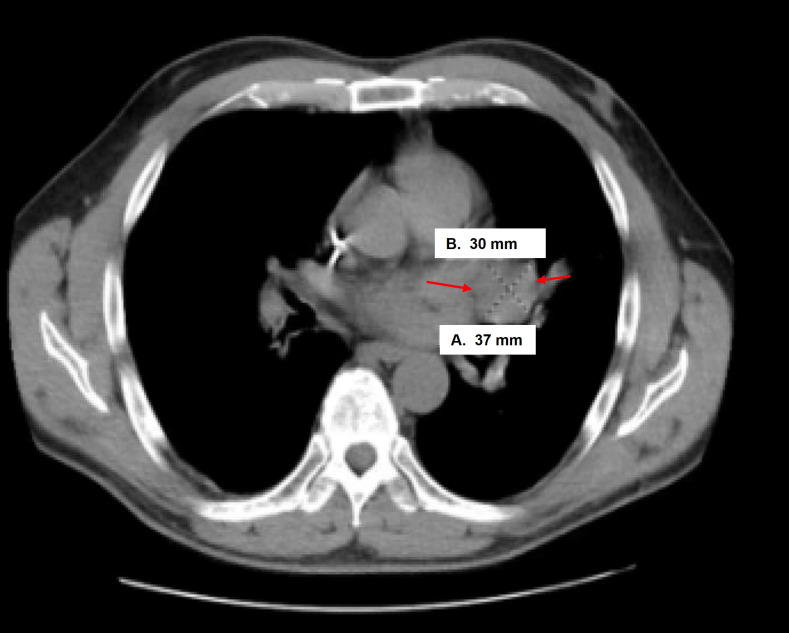
Computed tomography (CT) scan findings of clinical case 2 with enlarging hilar mass at 30 mm × 37 mm size (arrows)

Case 2 was a 58-year-old male who underwent biopsy of a left kidney tumor, which revealed HG UTUC. He then underwent left NU, which showed pT3NxMx UC with squamous differentiation of 40%. A re-staging computed tomography (CT) scan showed multiple lung nodules, a liver mass, and retroperitoneal (RP) adenopathy. A subsequent CT-guided liver biopsy showed benign liver tissue, but a follow-up re-staging scan identified a new left hilar mass (Figure 3) and enlarging lung nodules. An endobronchial ultrasound-guided fine-needle aspiration biopsy of the hilar mass confirmed metastatic HGUC. He was started on four cycles of gemcitabine and carboplatin, with a bronchial stent placement required due to worsening lung lesions. The patient transitioned to comfort care given lack of response to treatment.

### Clinical case 3

**Figure 4:**
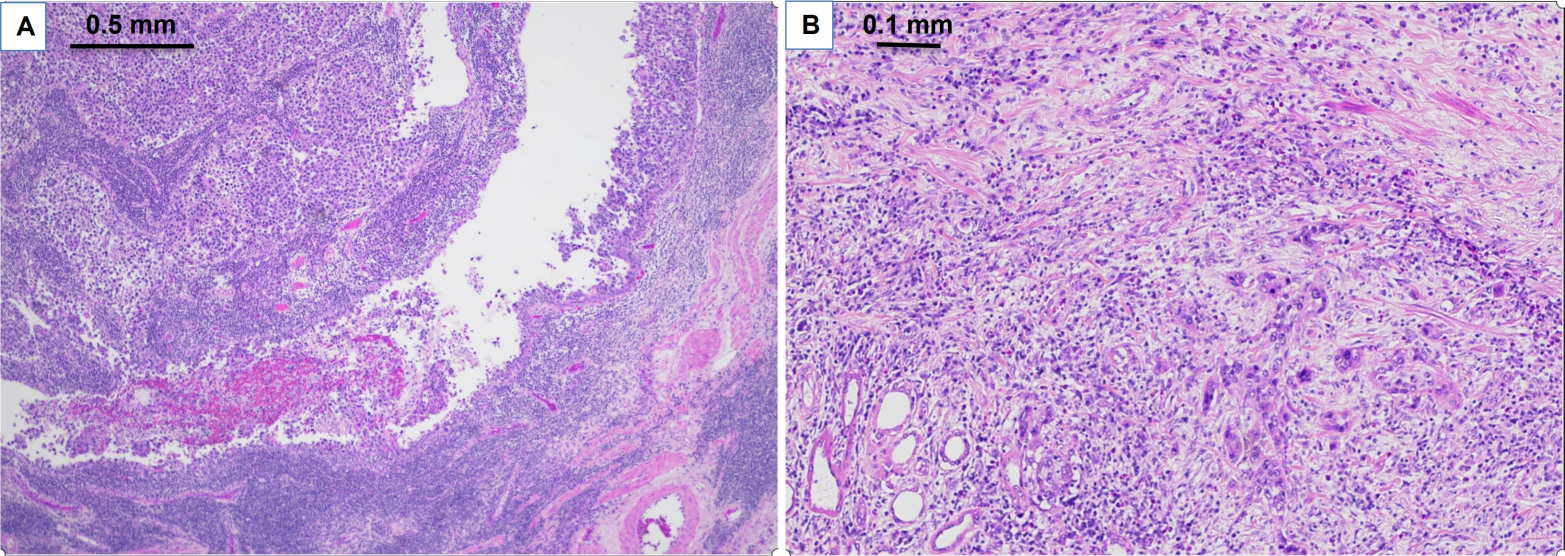
Pathologic findings on H&E for clinical case 3. A. Clinical case 3 pathological staging pT3 high grade papillary urothelial carcinoma of renal pelvis (40× H&E); B. high grade papillary urothelial carcinoma invades renal parenchyma (100× H&E)

**Figure 5:**
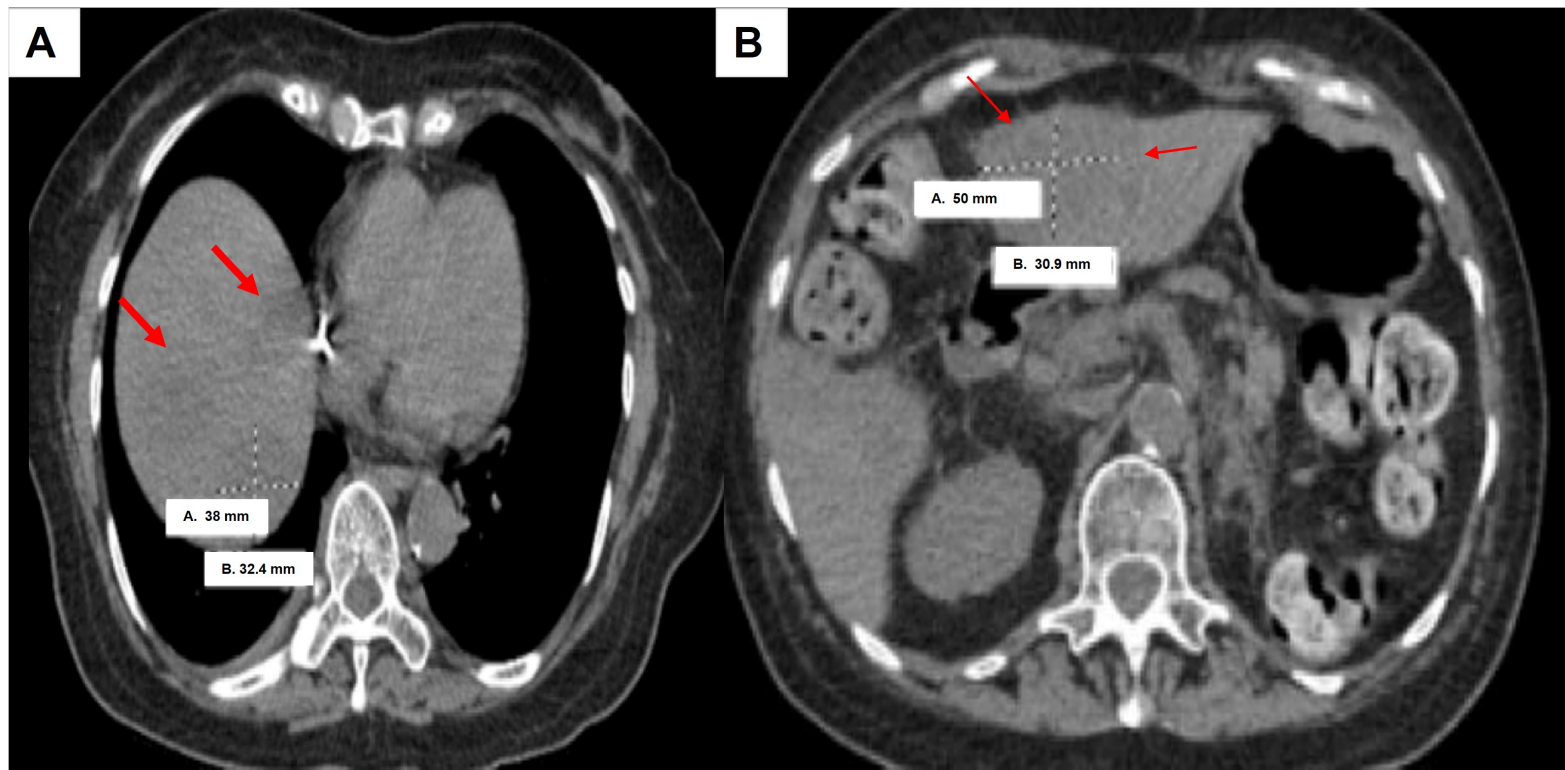
Computed tomography (CT) scan of clinical case 3 showing liver metastases (red arrows) and largest size measuring 38 mm × 32.4 mm

Case 3 involved an 80-year-old female who presented with a left renal pelvis mass and underwent left renal pelvis washing and biopsy, which showed HGUC. Further staging CT scan showed a small, non-specific 3 mm lung nodule. She then proceeded with left NU, which showed pT3pNxMx UC (Figure 4) with carcinoma in situ. She opted not to undergo adjuvant chemotherapy, but then subsequent re-staging CT showed prominent RP adenopathy, prompting the initiation of chemotherapy with gemcitabine and carboplatin for three cycles. However, due to worsening pain, another scan was performed which revealed an interval increase in RP and left periaortic adenopathy. Given these results, she was switched to pembrolizumab but only received two cycles before developing new liver metastases (Figure 5). This new development led to the patient’s eventual transition to hospice care.

### Clinical case 4

Case 4 is a 66-year-old male without significant prior history presented with a heterogeneously enhancing mass in the left renal pelvis with a biopsy showing papillary UC. He underwent a left radical NU, which showed pT3pN0Mx UC with 30% sarcomatoid features and 20% necrosis. Following surgery, he presented with back pain, and a subsequent scan demonstrated L3 lesion consistent with metastatic UC. He underwent an L3 partial vertebrectomy and laminectomy and received palliative radiation. He was then treated with six cycles of gemcitabine and carboplatin, but experienced worsening weakness and an enlargement of a soft tissue mass at T12, requiring additional palliative radiation. Despite these efforts, further progression of the disease led to his eventual transition to hospice care.

## Discussion

UTUC makes up only a fraction of cases of UC but tends to be more aggressive in its course [13]. Systemic therapy follows closely the treatment used in bladder cancers [1]. Decipher Bladder^®^ assay is a genomic subtyping tool that has been shown to predict response to NAC in muscle-invasive bladder cancers (MIBC) [6]. In addition, the standard treatment for UTUC involves surgery with NU in early stages for high-grade disease though NAC followed by surgery is frequently used in clinical practice [1]. Patients in this series all received chemotherapy, but only case 1 underwent NAC followed by NU. While all three other cases underwent NU upfront, all eventually got diagnosed with metastatic disease and received further platinum-based chemotherapy for metastatic disease but all cases quickly progressed thereafter.

The four cases presented in our series (Table 1) had mixed stages and clinical course. Only case 1 remained locally advanced stage (stage III) and underwent NAC with residual ypT3 disease after NU. Unfortunately, this patient succumbed to cardiovascular morbidity, resulting in a non-cancer-related death. The remaining three cases, none of whom have prior NAC exposure, quickly progressed to metastatic disease, and showed poor overall response to systemic therapy.

All four cases had basal molecular markers, although two patients had additional mixed claudin-low molecular subtype with increased stromal markers on the Decipher Bladder^®^ GRID (Figure 1). This led to their classification among the stroma-rich subtype by the consensus subtyping model [14]. Of interest, both case 1 and 4 had basal and basal/claudin-low molecular subtype but their clinical course and phenotype differed. Case 1 manifested residual ypT3 HGUC after NAC but remained non-metastatic, whereas case 4 showed a sarcomatoid component on immunohistochemistry. These factors likely portended poor outcomes regardless of chemotherapy treatment.

The co-occurrence of non-luminal subtype and sarcomatoid variant histology has been reported in other series where basal MIBC have been enriched with both squamous and sarcomatoid features, which can be associated with metastatic disease or advanced stage at presentation [15, 16]. Furthermore, low expression of keratin 5/6 (KRT 5/6) and KRT14 is also consistent with sarcomatoid transition with loss of epithelial markers [17]. While several mutations were notable in case 2, the rapid progression of the disease did not allow for any further switch in treatment.

The genomic characteristics of patients who undergo NU and their association with response to systemic chemotherapy in UTUC have not been studied extensively. In contrast, molecular markers in bladder cancer have been evaluated and suggested to predict both pathological upstaging at radical cystectomy and response to NAC. Non-luminal tumors have shown higher rates of pathological upstaging in the cystectomy-only setting without NAC [9] and were suggested to benefit most from NAC in a separate multi-institutional MIBC study [18]. Notably, all four cases within the present UTUC profiling study had a non-luminal subtype, with only one out of four cases being treated with NAC. Although none of these cases showed a convincing response, the aggressive behavior of non-luminal tumors was validated in the present study as reflected by high pathologic tumor stage. Moreover, whereas previous efforts on transcriptomic subtyping revealed many luminal-type tumors among UTUC [19], the present study solely included non-luminal tumors.

In addition to observing a luminal subtype in UTUC, Robinson et al. [19] described UTUC having a T-cell depleted contexture. In our study, two out of four non-luminal cases harbored substantial signal for the claudin-low subtype, a subtype previously described to have increased levels of immune signatures and suggested to benefit from systemic neoadjuvant pembrolizumab [20]. Lastly, although activated *FGFR3* signaling has been observed in UTUC [19], it was not evident in the present study when evaluating an *FGFR3* activity signature (Table 1) [21]. Furthermore, Foundation DNA analysis results did not show any *FGFR*-related DNA alterations. As such, both the non-luminal transcriptomic subtypes and lack of *FGFR*-related DNA alterations in the present case-series may represent UTUC cases with more aggressive biology, reflected by their clinical course associated with poor outcomes.

The initial goal of this small pilot project was to determine feasibility and pave the way for a future prospective trial to predict the response to NAC in UTUC. However, since the initiation of this project, additional studies, including the POUT trial, have demonstrated the benefits of adjuvant chemotherapy, revealing an improvement in three-year DFS in favor of adjuvant chemotherapy [2]. In addition, the CheckMate 274 trial showed that adjuvant nivolumab improved DFS, although the subgroup of UTUC patients who were capped at 20% enrollment had seemingly suboptimal responses with subgroup analyses hazard ratios for disease recurrence or death of 1.23 and 1.56 for renal pelvis and ureter, respectively [22].

Understanding the biologic aspects may help prioritize UTUC patients to certain types of systemic therapy in a prospective manner. It is important to note that this case series has several limitations, including the small retrospective nature, the wide heterogeneity of the population with mixed stages, and inconsistent availability of genomic sequencing platform other than the molecular subtyping.

In summary, our study evaluated the feasibility of obtaining molecular subtyping information from UTUC specimens and provided potential feasibility and increased understanding of genomics in UTUC. While a prospective trial may assess whether molecular biomarkers could predict response to NAC, it is yet unclear whether these studies could facilitate future sparing of completion NU in those who achieve complete or partial response. In addition, the real-world application of next generation sequencing as a tool to be used for clinical judgement in UTUC patients requires further clarification in a larger cohort study. Furthermore, the future role of biomarkers including ctDNA, may help further refine the molecular landscape of the UTUC patient population.

## Abbreviations

CT: computed tomography
DFS: disease-free survival
HG UTUC: high-grade upper tract urothelial carcinoma
HGUC: high-grade urothelial cancer
MIBC: muscle-invasive bladder cancers
NAC: neoadjuvant chemotherapy
NU: nephroureterectomy
RP: retroperitoneal
UC: urothelial cancer
UTUC: upper tract urothelial cancer

## Declarations

### Availability of data and materials

The raw data supporting the conclusions of this manuscript will be made available by the authors, without undue reservation, to any qualified researcher.

### Copyright

© The Author(s) 2024.
